# Supplementary material for: In Vitro Interaction of Binuclear Copper Complexes with Liver Drug-Metabolizing Cytochromes P450
Source: Pharmaceuticals (Basel). 2024 Sep 10;17(9):1194. doi: 10.3390/ph17091194 (PMC11434749; doi:10.3390/ph17091194)
Supplement: Supplementary file 1 [file pharmaceuticals-17-01194-s001.zip › pharmaceuticals-3173979-supplementary.pdf]

Supplement for

In vitro interaction of binuclear copper complexes with liver drug-metabolizing cytochromes P450

Alena Špičáková[1]*, Zuzana Horáčková[2], Pavel Kopel[3]* and Pavel Anzenbacher[1]

[1] Department of Pharmacology, Faculty of Medicine, Palacký University Olomouc, Hněvotínská 3, 779 00 Olomouc, Czech Republic; anzen@seznam.cz

[2] Laboratory of Growth Regulators, Faculty of Science, Palacký University & Institute of Experimental Botany of the Czech Academy of Sciences, Šlechtitelů 27, 78 371 Olomouc, Czech Republic; zuzi.horackova@seznam.cz

[3] Department of Inorganic Chemistry, Faculty of Science, Palacký University Olomouc, 17. Listopadu 1192/12, 779 00 Olomouc, Czech Republic

* Correspondence: alena.spicakova@upol.cz (A.Š.); pavel.kopel@upol.cz (P.K.); Tel.: +420-585632560 (A.Š.); +420-585634352 (P.K.)

Table S1. Crystal data and structure refinement for $[Cu_2(\mu\text{-fu})(pmdien)_2(H_2O)_2](ClO_4)_2$

Empirical formula	C22H52Cl2Cu2N6O14
Formula weight	822.67
Temperature/K	149.99(10)
Crystal system	monoclinic
Space group	P21/c
a/Å	8.0782(2)
b/Å	15.2754(4)
c/Å	14.4871(3)
α/°	90
β/°	102.268(2)
γ/°	90
Volume/Å^3	1746.85(7)
Z	2
ρcalcmg/mm^3	1.564
μ/mm^{-1}	3.518
F(000)	860.0
Crystal size/mm^3	0.25 × 0.23 × 0.19
2Θ range for data collection	8.516 to 136.604°
Index ranges	$-9 \le h \le 9$, $-17 \le k \le 18$, $-14 \le l \le 17$
Reflections collected	9555
Independent reflections	3165[R(int) = 0.0214]
Data/restraints/parameters	3165/0/215
Goodness-of-fit on F^2	1.038
Final R indexes [I>=2σ (I)]	R1 = 0.0307, wR2 = 0.0801
Final R indexes [all data]	R1 = 0.0323, wR2 = 0.0811
Largest diff. peak/hole /e Å^{-3}	0.38/-0.33

Table S2. Selected Bond Lengths for [Cu_2(μ-fu)(pmdien)$_2$(H_2O)$_2$](ClO_4)$_2$.

Length/Å	
Cu01 O1	1.9491(14)
Cu01 O2	2.2433(14)
Cu01 N2	2.0377(17)
Cu01 N1	2.0545(17)
Cu01 N3	2.0498(17)

Table S3. Selected Bond Angles for [Cu_2(μ-fu)(pmdien)$_2$(H_2O)$_2$](ClO_4)$_2$.

**Angle/°**	
O1 Cu01 O2	93.81(6)
O1 Cu01 N2	168.59(6)
O1 Cu01 N1	91.39(7)
O1 Cu01 N3	90.19(7)
N2 Cu01 O2	97.59(6)
N2 Cu01 N1	86.43(7)
N2 Cu01 N3	86.67(7)
N1 Cu01 O2	103.99(6)
N3 Cu01 O2	103.17(6)
N3 Cu01 N1	152.63(7)

The X-ray powder diffraction patterns were recorded on MiniFlex 600 X-ray difractometer (Rigaku, Austin, TX, USA) with Bragg-Brettano arrangement using CuK*α* radiation.

The composition of the prepared compounds was verified through powder X-ray diffraction.

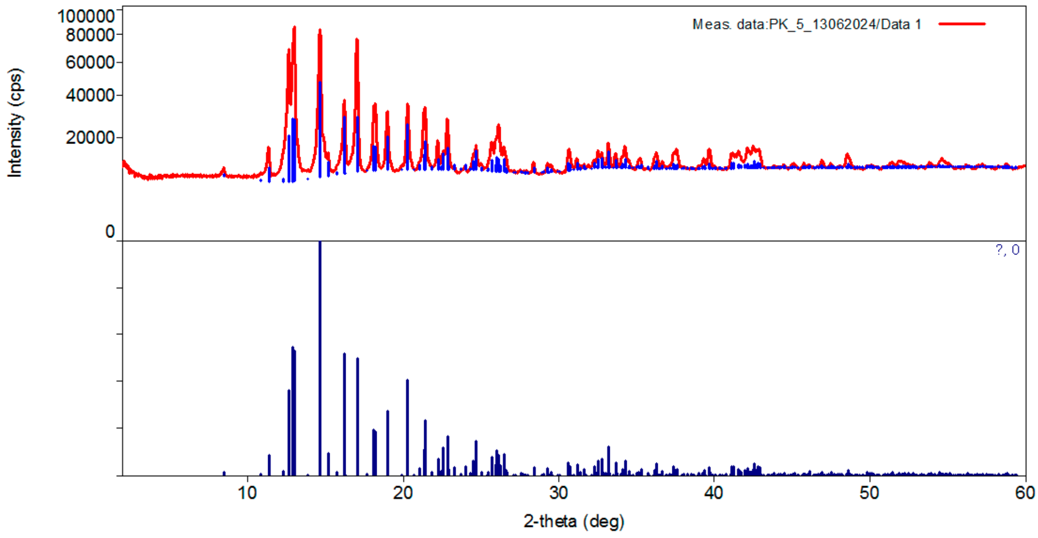

Figure S1A. Comparison of experimental X-ray powder diffraction pattern of complex No. 5 (red) measured at room temperature and diffractions calculated from single-crystal X-ray diffraction data (blue) measured at 298 K.

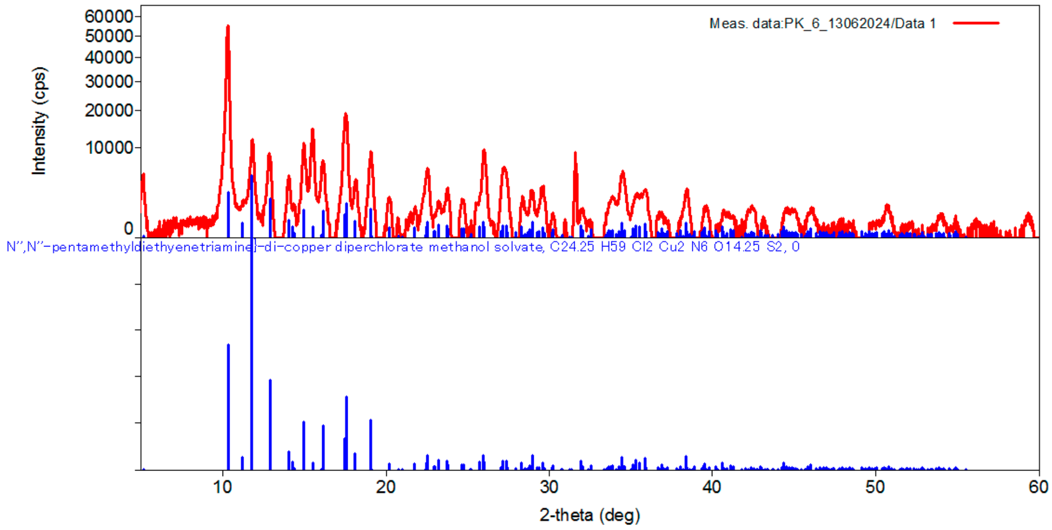

Figure S1B. Comparison of experimental X-ray powder diffraction pattern of complex No. 6 (red) measured at room temperature and diffractions calculated from single-crystal X-ray diffraction data (blue) measured at 298 K.

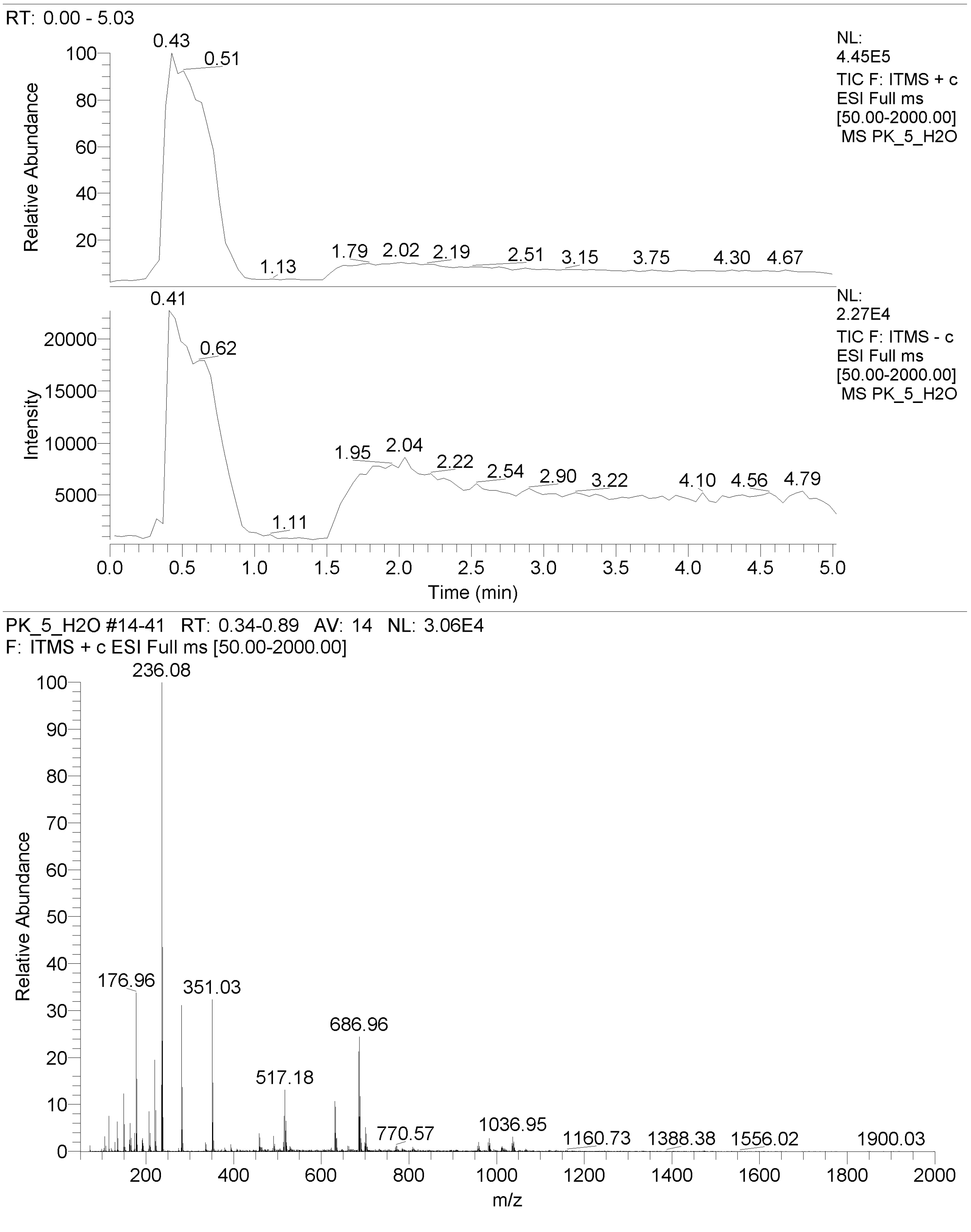

Figure S2A. The mass spectrum of complex No. 5 measured in water in the positive mode.

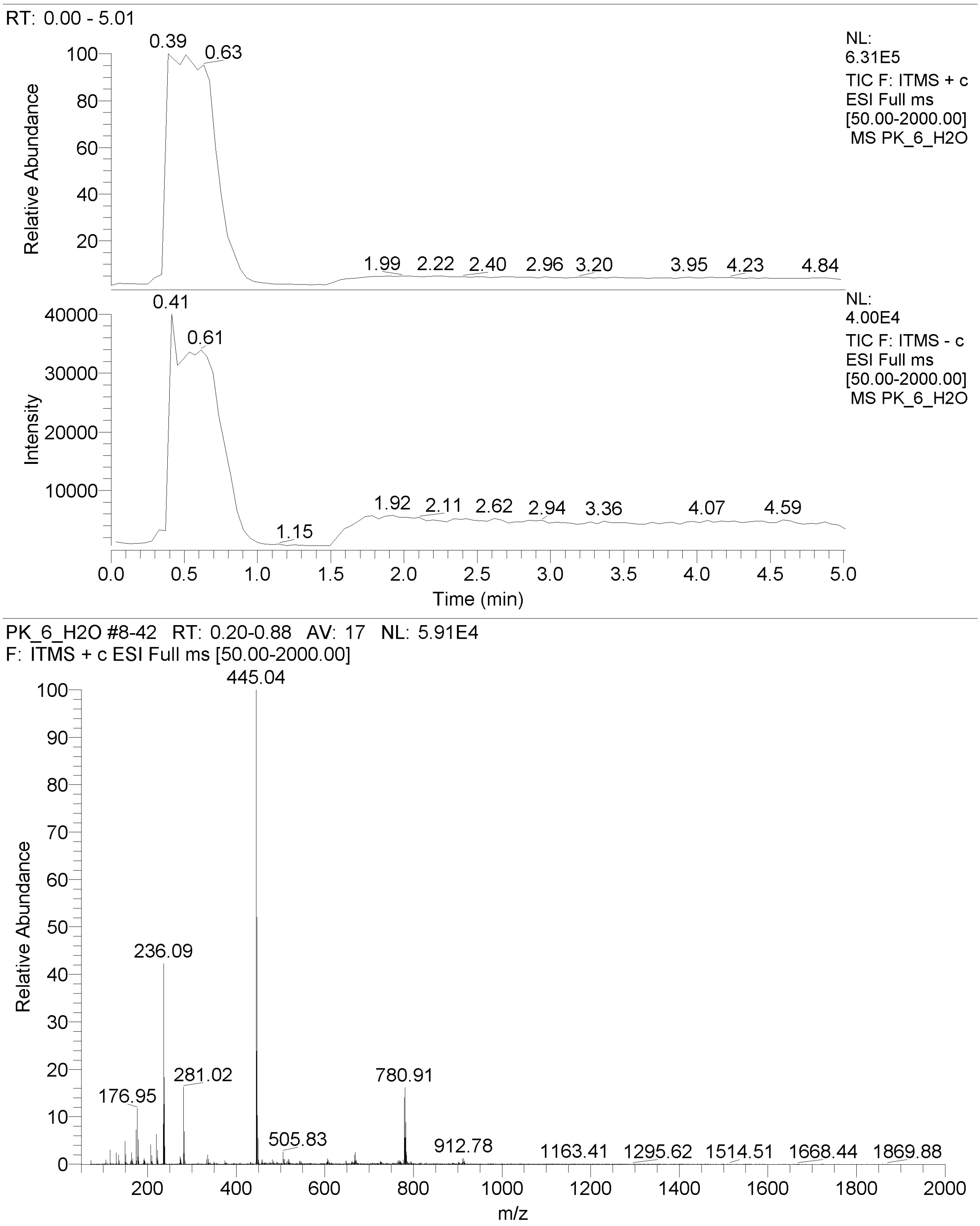

Figure S2B. The mass spectrum of complex No. 6 measured in water in the positive mode.

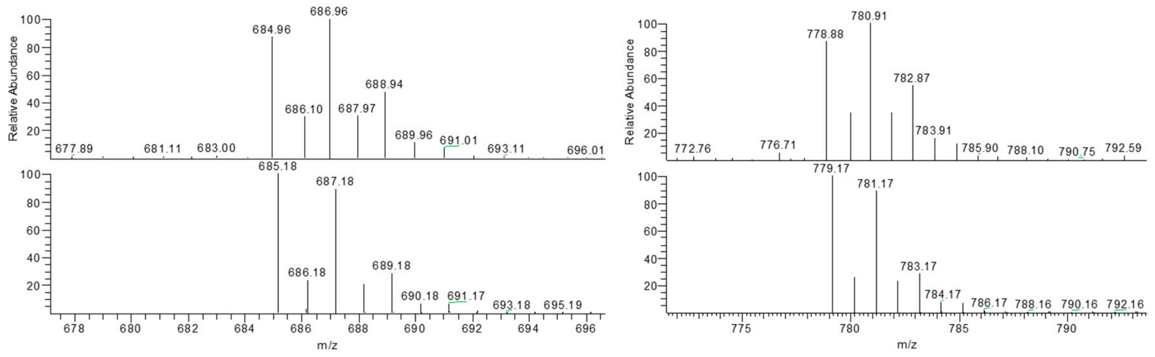

(a) (b)

Figure S3. A fragment of mass spectra with simulation patterns of complex ions No. 5 $[Cu_2(fu)(pmdien)_2(ClO_4)H^+]^+$ (a) and No. 6 $[Cu_2(dtdp)(pmdien)_2(ClO_4)H^+]^+$. Measured spectra are on top and calculated at the bottom of the figures.
